# Atmospheric Resuspension of Microplastics from Bare Soil Regions

**DOI:** 10.1021/acs.est.4c01252

**Published:** 2024-05-20

**Authors:** Ioanna Evangelou, Daria Tatsii, Silvia Bucci, Andreas Stohl

**Affiliations:** Department of Meteorology and Geophysics, University of Vienna, Universitätsring 1, Vienna 1010, Austria

**Keywords:** plastics, mineral dust, soil, enrichment ratio, fiber, atmospheric transport

## Abstract

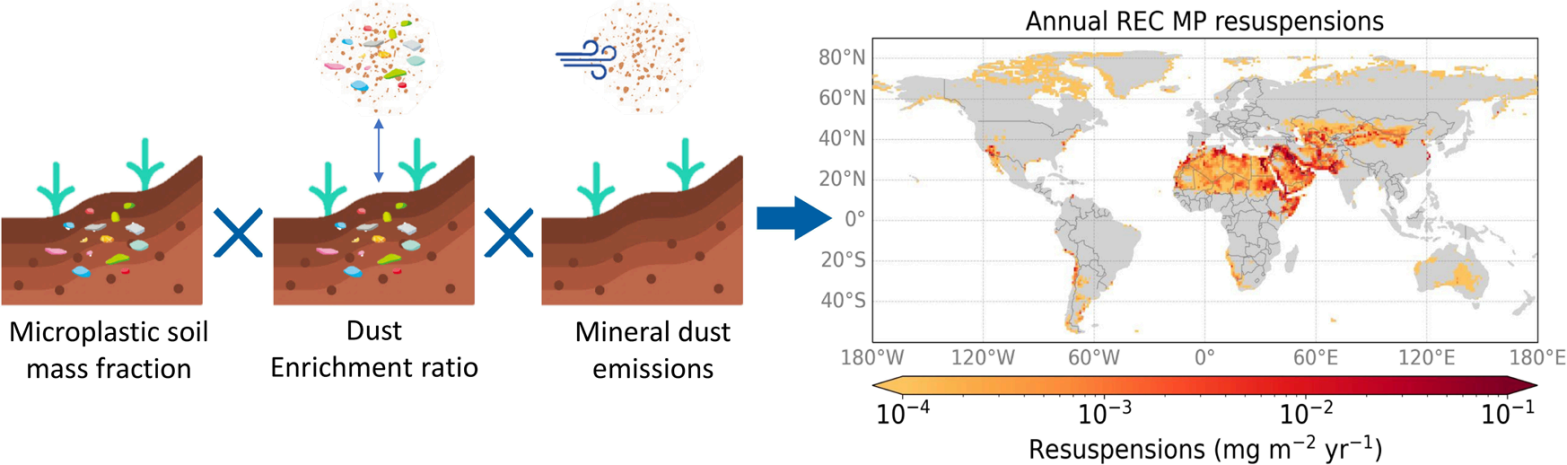

Microplastics (MPs) are emerging as an atmospheric pollutant. Here, we present a method of estimating MP resuspension with mineral dust in bare soil based on reported MP mass in soils, their enrichment in suspended dust relative to soil, and a mineral dust resuspension scheme. Using the estimated resuspensions, we simulate the global atmospheric MP transport and deposition using the dispersion model FLEXPART for two particle shape scenarios, spheres, and fibers. We estimate the uncertainties using a Monte Carlo technique that varies input data parameters within their reported ranges. The total MP resuspensions are estimated at about 104 (48–110) tonnes yr^–1^. We find that bare soils in West Asia and North Africa are the main source regions. FLEXPART results show that fibers have higher concentrations in the atmosphere and are dispersed more widely than spheres. Annually, 75 (43–83) tonnes of microfibers are deposited on land and 29 (18–33) tonnes in the oceans. Resuspended MPs can even reach remote regions, such as the Arctic. The results suggest that areas with bare soils can be an important MP source; however, further research on the factors that affect resuspension is needed.

## Abstract 2

Microplastic resuspensions from bare soils are not extensively studied. This study develops a resuspension estimation method and reports that the emitted microplastics can be dispersed globally, affecting all environmental compartments.

## Introduction

Microplastics (MPs) are synthetic polymer particles with sizes between 1 μm and 5 mm, produced intentionally or resulting from the weathering of larger plastic particles.^1^ In the environment, MPs can be encountered in different sizes, polymer types, colors, and shapes (fibers, fragments, spheres, etc.).^2^ MPs have been found in soils and sediments,^3^ freshwater and oceans,^4^ wastewater and sewage sludge,^5^ as well as in plants,^6^ animals,^7^ and humans.^8^ Microplastics are also present in the atmosphere and contribute to air pollution. Particles of such small size can be transported over long distances, with the potential to reach remote regions on Earth, including pristine regions such as the Arctic.^9^ They can adsorb and carry toxic substances^10^ and may also change the Earth’s energy balance.^11^

Despite the importance of the atmosphere for global MP dispersion, research regarding MPs in the atmosphere is lagging behind. Only relatively small particles can be suspended in the atmosphere, and unambiguous detection of such small MP particles is still difficult, if not impossible. Airborne MPs result from human use of plastics such as car tires and brakes worn during driving, abrasion of other plastic products, landfills, or contaminated agricultural fields.^9,12−14^ MPs are transferred from the atmosphere via wet and dry deposition to terrestrial and marine environments, where they pose a threat to ecosystems and possibly act as a further secondary MP source for the atmosphere. For instance, MPs can be injected into the atmosphere by sea spray from the polluted ocean^4^ and resuspension from soils.^15^ Brahney et al.^16^ and Evangeliou et al.^17^ estimated the emissions from different source types using an optimization method based on measurements in western North America. Results indicated that atmospheric MPs are mostly originating from secondary sources.

Mineral dust is one of the most important aerosols in the Earth’s atmosphere, as it is a main contributor to the total global aerosol mass burden.^18^ Emissions of mineral dust can be natural or anthropogenic, based on the source regions.^19^ Anthropogenic dust emissions are connected with wind erosion of lands that are used for agricultural practices or with other human activities, such as road dust, while natural dust emissions originate from bare and erodible soil surfaces. In such regions, MPs contained inside or outside soil aggregates can be resuspended by the wind together with the natural dust. The size distribution of MPs in emitted dust can be affected by processes such as saltation and creep.^20^ Bullard et al.^21^ have investigated the fragmentation of MP beads by quartz particles due to saltation for different wind abrasion time periods. They found that MPs can lose 80% of their mass and some 50% of their diameter after 300 h of aeolian abrasion. Nonspherical particles experience a larger drag than spherical particles of the same volume and consequently have a longer atmospheric lifetime.^22^ It is therefore important to also take into account the shapes of resuspended particles to correctly capture the atmospheric transport processes.

To date, only a few estimates of MPs co-emitted with mineral dust from natural bare, erodible soils are available. The purpose of this study is to develop a bottom-up method to estimate these resuspensions based on published measurements of MP mass fraction in soils, their reported enrichment in resuspended dust, and a mineral dust emission scheme. We provide the first estimates of MP resuspensions from bare soils and bracket their uncertainty by employing Monte Carlo (MC) simulations. Finally, we simulate the atmospheric transport of resuspended MPs to obtain the global concentration and deposition fields of resuspended MPs originating from bare soils.

## Materials and Methods

### Estimation of Microplastic Resuspensions

In order to estimate the resuspension flux of MPs from bare soils, *F*_MP_, we use the following equation
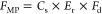
1where *C*_s_ is the mass fraction of MPs in soil (i.e., the mass of MPs divided by the mass of dry soil), *E*_r_ is the enrichment ratio of MP mass fraction in resuspension relative to MP mass fraction in the soil, and *F*_d_ is the flux of mineral dust. The motivation for this approach is to take advantage of the comprehensive process understanding of mineral dust emissions that has been obtained in the past decades.^23^ In the following, we describe how each of the factors in eq 1 is determined. We first explain how we made our best estimate of MP resuspension fluxes and subsequently describe the propagation of uncertainties.

#### Microplastic Mass Fractions in Soils

**Figure 1:**
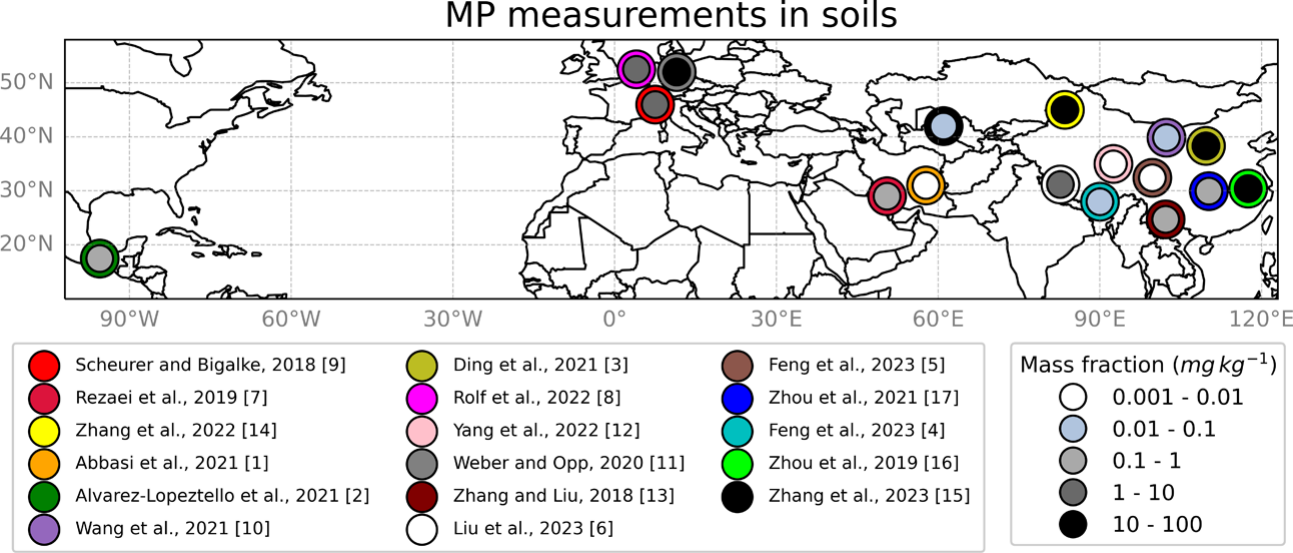
Locations for which measurements of MP soil mass fraction were available, with the small dots corresponding to the measured mass fraction and the large colored dots underneath referring to the respective studies. Average measured values are reported in Table S1. The studies referenced can be found in the Supporting Information.

The observed range of MP content in various types of soil covers orders of magnitude. Municipal regions, for instance, can have ten times higher MP soil mass fractions than rural areas.^3^ We use measurements from 17 studies (in total 185 measurement points), listed in Table S1, that report MP soil mass fractions in sandy regions, vacant lands, and very sparsely vegetated soils (see Figure 1 and Table S1). We strictly exclude all data taken in agricultural areas. For studies reporting MP fractions as a number of MP particles per mass of dry soil, we converted the data to MP mass fractions using the most common shape and size of the particles reported by the respective study and an MP density of 1050 kg m^–3^ (Table S1). This is a typical density for common plastic materials.^24^

**Figure 2:**
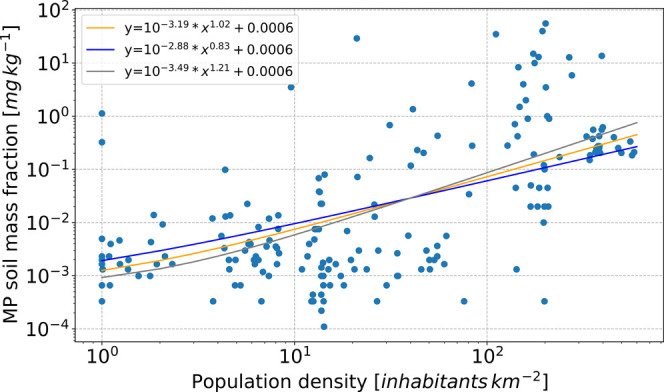
MP soil mass fraction measurements and corresponding population density for the radius of 300 km. The best fit (orange line) used for the reference emission case (REC) resuspensions as well as the 2σ uncertainties (blue and gray) are depicted.

As the use of plastics is directly connected with human activities, it is reasonable to assume that the MP content in soils is related to the population density in the surrounding area. Therefore, we investigated how the measured MP soil mass fractions are correlated to population density. In order to assign a population density to each measurement, we used a  resolution population map.^25^ Since the spatial scale of human impact on MP soil content is not clear, we calculated the average population density in circular areas with radii of 10, 30, 50, 100, 150, 200, 250, 300, 500, and 1000 km around each measurement location. We then calculated the linear correlation coefficient between the logarithm of the measured MP soil mass fractions and the logarithm of the average population density. The highest value (*r* = 0.61) was found for the spatial scale of 300 km, so we use this radius as the best estimate for the resuspension calculations. The MP soil mass fraction as a function of population density with the 2σ uncertainty for the linear regression slope and intercept is shown in Figure 2. We have also added a minimum intercept based on the 10th percentile of the measured MP soil mass fractions (0.0006 mg kg^–1^), as zero population density does not correspond to zero MP soil mass fraction. We subsequently used the population map to obtain a 0.5° × 0.5° inventory of MP soil mass fractions in bare soil regions. Another approach of getting the spatial distribution of soil MPs is the method of Brahney et al.,^16^ who simulated the total deposition originating from population-related primary emissions and then combined these with dust emissions. For this method, however, knowledge of primary emissions is needed, a source we do not study here and which is still not well estimated. It is also not clear whether atmospheric transport is the major pathway of MP to bare soil regions since humans are passing through these regions (e.g., traffic, recreation) and may contaminate the soils directly.

#### Enrichment Ratio

Several studies have shown that airborne MPs in resuspended dust are enriched compared to MPs in the soil.^15,26,27^ This is likely due to the lower density of plastics compared to mineral dust and is probably also related to their often more complex shapes, both of which enhance resuspension.^28^ Values for the number enrichment ratio (ER), i.e., the ratio of MP number fraction in the airborne wind-eroded sediment (in particles/kg) to the MP number fraction in the primary soil (in particles/kg), were determined in a wind tunnel by Bullard et al.^26^ Since all MP particles used in Bullard et al.^26^ have the same density and volume, their number ER corresponds to the ER defined in eq 1. They found that ER values depend on the MP soil mass fractions and range from 1.06 to 3.49. Our MP soil mass fractions correspond best to their ER value of 3.49. We also use the alternative ER value of 2.11 based on measurements in a dried river bed and rangelands^15^ for estimating the impact of ER uncertainty.

#### Mineral Dust Emissions

To determine the third factor in eq 1, we calculated global, 3-hourly, 0.5° × 0.5° resolution mineral dust fluxes for the year 2018 using the dust mobilization scheme FLEXDUST.^29^ FLEXDUST calculates the dust emission from bare soils (unconsolidated materials) and the sparsely vegetated land (sparse herbaceous vegetation, sparse woody vegetation) from the Global Land Cover by National Mapping Organizations (GLCNMO).^30^ For the latter, the bareness of the land is calculated by subtracting the vegetation cover fraction used by the European Centre for Medium-Range Weather Forecast (ECMWF). Land cover classes, such as the Cropland and Vegetation Mosaic classes of GLCNMO, are not included; thus, agricultural areas are not included in the calculation of the mineral dust emissions. The erodibility scaling factor of Ginoux et al. 2001^31^ is applied to the bare soil fraction to get the erodible part of the land. The dust flux calculation is done with the emission model MB95^32^ and the volume size distribution of Kok 2011.^33^ The friction velocity used for the flux estimation relies on ECMWF shear stress data. As meteorological input to FLEXDUST, we used ECMWF ERA5 global meteorological fields at 0.5° × 0.5° horizontal and 1 h temporal resolution.^34^

Applying eq 1 and thus multiplying the spatially varying MP soil mass fraction with the constant ER value of 3.49 and the 3-hourly, 0.5° × 0.5° resolution dust emissions, we obtain the spatially and temporally resolved MP resuspensions. We refer to this basic resuspension scenario as the reference emission case (REC) from this point on. The REC resuspensions are regridded to a 1° × 1° horizontal resolution.

### Resuspension Uncertainties

To quantify the uncertainties of our resuspension estimates, we employ MC simulations. MC simulations are a statistical approach that relies on a repeated random sampling of the input variables of a deterministic problem in order to obtain multiple numerical solutions characterizing the output uncertainty. Here, we use the MC simulation case of bootstrapping,^35^ which is valuable when the underlying probability distribution of the input data is unknown. After the MC simulation, the uncertainty of the evaluated metric can be estimated with error statistics such as the standard deviation.

We conduct a ten-thousand-member MC simulation perturbing the factors that most probably affect our resuspension estimations: the MP soil mass fraction, the spatial scale used for calculating the population density, and the ER value (see eq 1). The uncertainties associated with the dust emissions provided by FLEXDUST are considered smaller than the others and are not accounted for in the simulation. We use ten variations for the spatial scale and two for the ER (3.49 and 2.11). Three variations are used for the MP soil mass fraction, the linear regression relation, and the ±2σ errors of it. In total, we have 60 possible combinations. In each iteration of the MC simulation, we randomly select one case of the 60 with a replacement. From the resulting ten thousand resuspension variations calculated as described in eq 1, we determine statistical parameters such as the mean  and the standard deviation (σ), as well as the geometric mean (μ_g_) and the geometric standard deviation (σ_g_).

### Simulations of the Atmospheric Transport of Microplastics

We use the Lagrangian particle dispersion model FLEXPART (FLEXible PARTicle dispersion model)^36^ version 11 [Bakels et al. (in preparation)] to simulate the atmospheric transport of resuspended MPs. The model is driven by global ECMWF ERA5 hourly meteorological data with 0.5° × 0.5° resolution for the year 2018. Output with a 1° × 1° resolution is produced every 6 h. Global MP REC resuspensions are put in FLEXPART with 1° × 1° horizontal and 6-hourly temporal resolution. We simulate the global atmospheric transport of MPs of two shapes (spheres and fibers) and eight different sizes (0.1, 0.5, 1, 2, 5, 10, 15, and 35 μm of volume equivalent diameter). Fibers are assumed to be cylinders with an aspect ratio of 50 (i.e., their length is 50 times their diameter), which is a typical ratio for fibers^37,38^ and with the same volume as the eight size classes used for the spheres. The gravitational settling for fibers is calculated with the scheme of Bagheri and Bonadonna,^39^ as modified and implemented into FLEXPART by Tatsii et al.^40^ The settling velocities of fibers with an aspect ratio of 50 using the Bagheri and Bonadonna^39^ scheme are very similar to those from Xiao et al.^41^ It is assumed that the orientation of fiber in the atmosphere is an average between random and maximum-drag (equivalent to the maximum projection area facing downward) orientation. The density of particles is set to 1050 kg m^–3^.

MP particle trajectories are terminated two months after their release^36^ to reduce the computational cost of the simulation. This corresponds to several times the typical lifetime of even the smallest simulated particles in the atmosphere and thus has virtually no effect on simulation results. We set cloud condensation nuclei and ice nuclei efficiencies of MPs to values of 0.05 and 0.15, respectively, the medium efficiency values reported by Grythe et al.^42^

**Figure 3:**
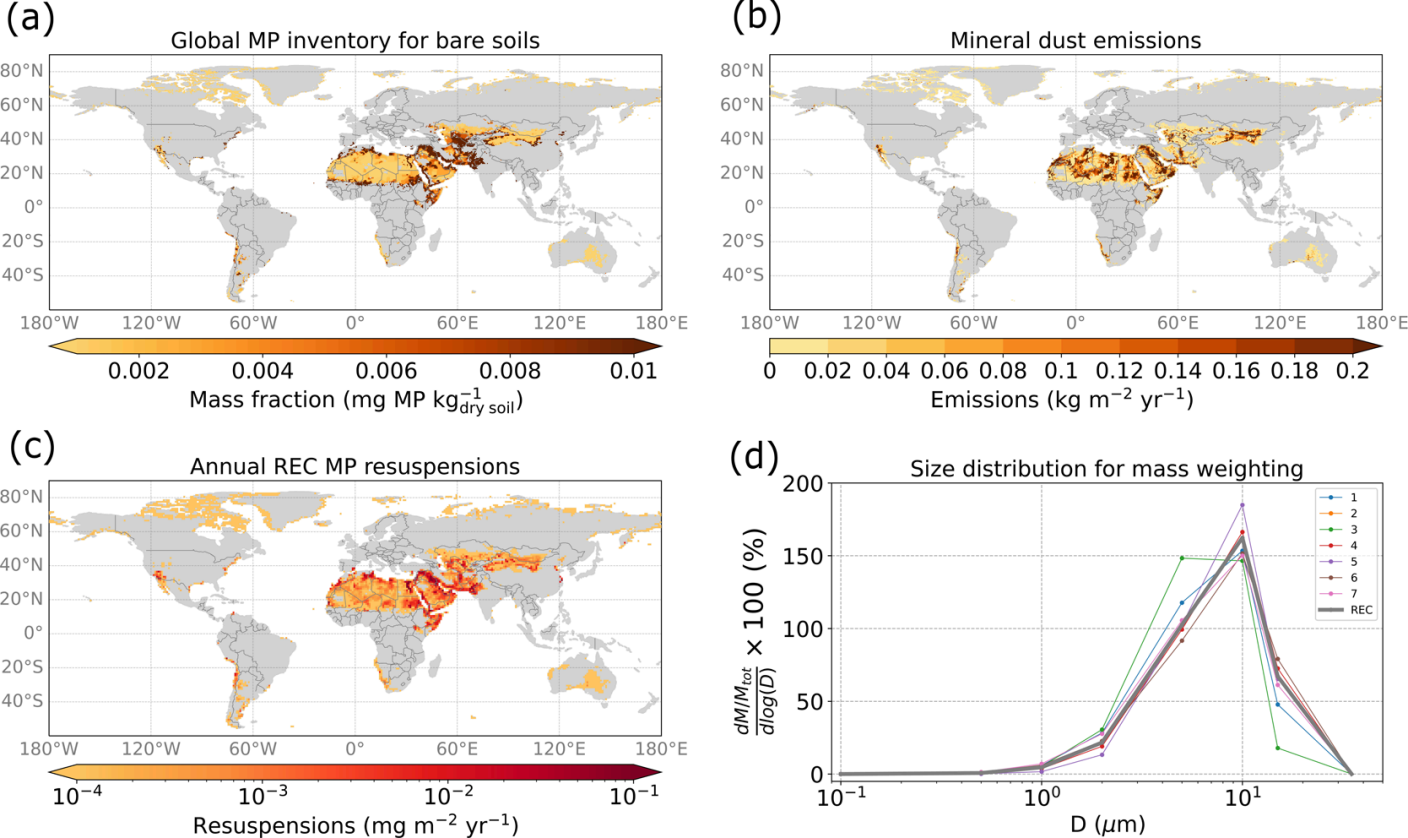
REC resuspension estimation for the year 2018. MP soil mass fractions (a), annual mineral dust emission fluxes (b), annual REC MP resuspension fluxes (c), and size distributions used for the MP transport simulations (d). The thick line in (d) corresponds to the REC case, and the thin lines show variations based on the uncertainty of the parameters in the Kok (2011)^33^ volume size distribution.

To our knowledge, no size distributions for MP particles smaller than 35 μm have been reported for MP resuspension from bare soils. For our REC simulations, we assume that, to some extent, the abrasion and fragmentation of MPs resemble those of mineral dust. We, therefore, base the emitted particle mass size distribution (thick line, REC in Figure 3d) on the volume size distribution of Kok,^33^ which is also used in FLEXDUST.

### Uncertainties of Atmospheric Transport Calculations

Uncertainties in the simulated atmospheric MP concentrations and deposition fields result from the resuspension uncertainties already dealt with, from the assumed emitted size distribution and shape of MPs, and from the transport model calculations. For estimating the uncertainty, we conduct extended MC simulations by varying both the resuspension estimates and the particle size distribution. We use six resuspension scenarios, the two variations for the ER and the three variations for the MP soil mass fraction of the 300 km case. We use the REC size distribution and seven variations of it (Figure 3d); thus 6 × 8 = 48 different possible combinations, and again apply bootstrapping to calculate a ten-thousand-member MC ensemble. Uncertainties in the simulated transport are assumed to be relatively smaller and are thus not included in the MC simulations. However, we conduct separate simulations for spheres and for fibers to explore the impact of particle shape on the simulation results.

## Results and Discussion

### Microplastic Resuspensions from Bare Soils

Remote areas of the Arctic, Australia, and parts of the Saharan desert (Figure 3a) contain less than 0.002 mg MP kg^–1^ of dry soil. Populated regions found in the United States and at the fringes of the main deserts show higher contamination of MPs, reaching 6 mg MP kg^–1^ of dry soil.

Regarding the mineral dust emissions (Figure 3b), the global annual emissions calculated by FLEXDUST for the year 2018 are approximately 2200 × 10^6^ tonnes yr^–1^, with the majority of the emissions taking place in North Africa, Saudi Arabia, and southwest Asia, a little higher than the estimation of 1600 × 10^6^ tonnes yr^–1^ obtained by Groot Zwaaftink et al.^29^ with FLEXDUST for the years 2010–2012 and similar to other estimates of global mineral dust emissions.^23^

The total annual MP resuspensions for different geographical regions are reported in Table 1 as total REC resuspensions together with the MC *x̅* and σ, as well as the MC μ_g_ and σ_g_. In the text, we report REC resuspensions together with an uncertainty range in parentheses spanning from the value of μ_g_/σ_g_ to the value of μ_g_ × σ_g_. The total annual REC MP resuspensions (Figure 3c) are estimated to be about 104 (48–110) tonnes yr^–1^. West Asia and North Africa are the biggest MP resuspension sources, with 57 (25–58) tonnes yr^–1^ and 40 (18–42) tonnes yr^–1^, respectively, as reported in Table 1. Note that mineral dust emissions are 39% higher in Africa than in Asia. Thus, the higher MP resuspensions in Asia are a result of the larger contamination of bare soils there. East Asia and North America are also noteworthy MP resuspension contributors, while resuspensions from Europe, Russia, and Oceania are much smaller—a consequence of relatively small mineral dust emissions in these regions. Resuspensions of MPs are particularly high in the Middle East, where both population density and mineral dust emissions are high.

**Table 1:** Total Annual MP Resuspensions for the Bare Soils Globally and for Different Continental Regions (Figure S1)^a^

region	REC resuspensions (tonnes yr^–1^)	*x̅* (tonnes yr^–1^)	σ (tonnes yr^–1^)	μ_g_ (tonnes yr^–1^)	σ_g_
total global land surface	104.4	78.0	27.1	72.4	1.5
West Asia	57.3	41.0	14.5	37.9	1.5
North Africa	40.1	30.0	10.7	27.8	1.5
East Asia	3.7	3.1	1.3	2.9	1.6
North America	1.6	2.2	0.8	2.0	1.6
South America	1.1	1.1	0.6	0.9	2.0
Europe	0.38	0.29	0.11	0.27	1.5
South Africa	0.14	0.16	0.12	0.13	2.2
Russia	0.07	0.07	0.04	0.06	1.9
Oceania	0.04	0.02	0.02	0.02	1.8
Greenland	0.0007	0.0007	0.0004	0.0006	1.6

a REC resuspensions, as well as the MC mean  and standard deviation (σ), and the geometric mean (μ_g_) and geometric standard deviation (σ_g_) of resuspensions are reported.

Dust emissions show a seasonal variation in both hemispheres.^43^ Thus, the MP entrainment is expected to follow a seasonal pattern as well. The global REC resuspensions are 24 tonnes for December, January, and February (DJF), 28 tonnes for March, April, and May (MAM), 44 tonnes for June, July, and August (JJA), and 20 tonnes for September, October, and November (SON). For the major MP emitters of North Africa and West Asia, JJA is the season with the highest total resuspensions (13 and 29 tonnes, respectively) (Table S2).

To date, no bottom-up estimates of MP resuspensions from bare soil regions specifically exist; however, MP resuspension with mineral dust from bare soils is probably lower compared to other emission sources. Previous studies^16,17^ estimated MP resuspensions indirectly via inverse modeling, although based on measurements in only one specific region (North America). Brahney et al.^16^ calculated around 70,000 (0–400,000) tonnes yr^–1^ of MPs emitted with dust originating from croplands, assuming that all agricultural fields have the same MP content. Evangeliou et al.^17^ reported that agricultural MP resuspensions are 310,000 tonnes yr^–1^ and resuspensions from the traffic sector are 280,000 tonnes every year, values that are orders of magnitude larger than our resuspensions. The agricultural sector emission values are not directly comparable to our values since we only consider resuspensions in natural bare soil regions. Brahney et al.^16^ also calculated the MP resuspensions from bare soils in natural arid lands, giving a global estimate of 18,000 tonnes yr^–1^, and Evangeliou et al.^17^ found resuspensions from dry land of about 30,650 tonnes yr^–1^ for sizes of 5–25 μm, using FLEXDUST for extrapolation, assuming that the emissions of this sector are 26% of the global MP emissions. These values are about one hundred times higher than our bottom-up estimate. However, their estimates are not constrained by physical process quantification but rather represent a fit to the measurement data in a single nonarid location. Consequently, we suspect that these values are strong overestimates of true emissions.

In agricultural regions, mineral dust emissions are much lower than in bare soil regions. For instance, Tegen et al.^44^ estimated that agricultural emissions contribute less than 10% of global dust emissions, while Ginoux et al.^45^ estimated that 25% of the global dust emissions are anthropogenic. Chen et al.^19^ reported that 19% of global dust emissions originated from indirect and direct anthropogenic sources. While our resuspensions are only from bare soil regions, these include many important dust source regions that are classified in these studies as agricultural (e.g., the Sahel). For these regions to dominate the global MP resuspensions and explain the high values obtained by Brahney et al.^16^ and Evangeliou et al.^17^ would, therefore, require MP soil mass fractions that are orders of magnitude higher than our estimate. For instance, fields treated with contaminated sewage sludge^5^ or plastic mulch^46^ may represent emission hot spots when resuspension occurs naturally or is triggered by agricultural practices. It is beyond the scope of the present study to estimate MP resuspension from such localized and likely sporadic sources. Determining bottom-up MP emissions from agricultural fields will require globally representative measurements of the MP soil content, as well as detailed information on agricultural practices and dust resuspension from such fields. Our estimates of MP resuspension from bare soil regions are, therefore, likely lower estimates of all global MP resuspension emissions. However, on the basis of our study, it is difficult to understand the very high values obtained by Brahney et al.^16^ and Evangeliou et al.,^17^ which may be more representative for the agricultural emission fluxes in the United States. These regions are also highly populated in our study, and extrapolating MP resuspension from these regions to the globe without accounting for differences in MP soil content would lead to much higher emission fluxes than we report here.

### Atmospheric Concentrations and Deposition of Resuspended Microplastics

**Figure 4:**
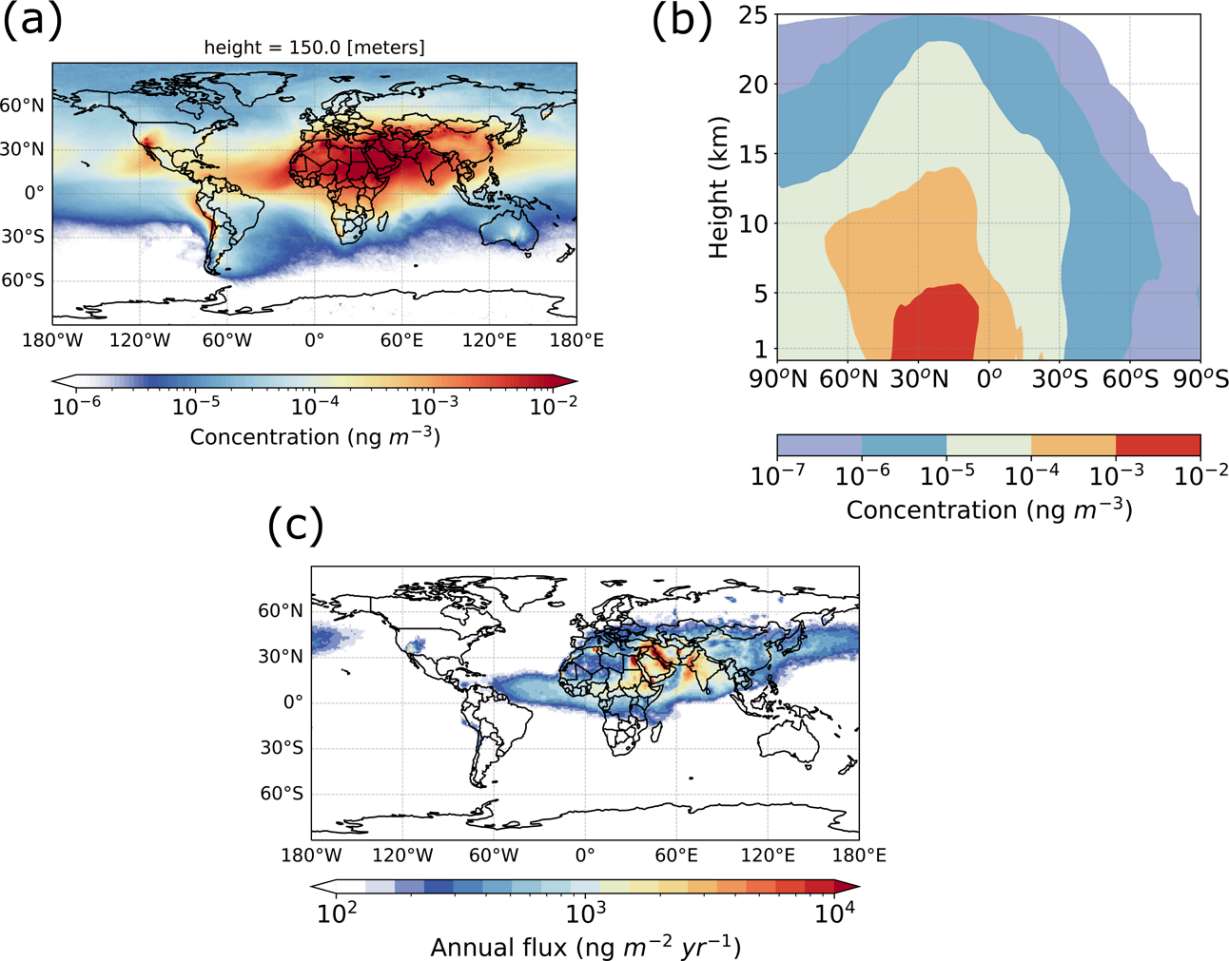
Atmospheric MP concentrations and deposition fluxes simulated by FLEXPART for the REC case for the year 2018. (a) Near-surface concentrations (0–150 m agl) and (b) zonally averaged concentrations as a function of latitude and altitude when assuming that the emitted MP particles are fibers. (c) Sum of simulated wet and dry deposition fluxes of MP fibers for the year 2018.

Annual average atmospheric near-surface concentrations of resuspended MP fibers and their zonally averaged values as a function of latitude and height, as well as their annual MP deposition values obtained from FLEXPART transport simulations, are depicted in Figure 4. Results for spheres as well as for seasonal averages for both spheres and fibers are given in the Supporting Information. The MC uncertainty for the annual average concentrations and for the annual total deposition flux are reported in Tables 2 (fibers) and S3 (spheres), and Tables 3 (fibers) and S4 (spheres), respectively.

**Table 2:** Annual Mean MP Near-Surface (0–150 m agl) Concentrations Averaged Over Different Continental Regions When Assuming That the Emitted Particles are Fibers^a^

region	REC^b^ (pg m^–3^)	*x̅* (pg m^–3^)	σ (pg m^–3^)	μ_g_ (pg m^–3^)	σ_g_
West Asia	20.0	17.2	5.9	16.3	1.40
North Africa	9.3	8.3	3.1	7.8	1.44
East Asia	1.5	1.3	0.4	1.3	1.37
South Africa	0.45	0.40	0.13	0.38	1.38
Europe	0.38	0.33	0.09	0.32	1.34
South America	0.30	0.25	0.07	0.24	1.30
North America	0.14	0.12	0.04	0.11	1.37
Russia	0.10	0.09	0.03	0.09	1.33
Greenland	0.021	0.020	0.008	0.018	1.47
Oceania	0.017	0.015	0.004	0.014	1.35
Antarctica	0.0005	0.0004	0.0002	0.0004	1.44

a REC concentrations as well as the MC mean and the standard deviation, the geometric mean and geometric standard deviation are reported.

b Reference Emission Case.

**Table 3:** Same as Table 2 but for Annual Total (Wet and Dry) Deposition

region	REC (tonnes)	*x̅* (tonnes)	σ (tonnes)	μ_g_ (tonnes)	σ_g_
total global land surface	74.8	63.1	20.7	59.9	1.38
total global ocean surface	29.1	25.5	8.3	24.2	1.38
West Asia	29.5	24.2	8.1	22.9	1.40
North Africa	22.8	19.4	6.9	18.3	1.42
East Asia	13.9	12.0	3.8	11.5	1.37
Russia	2.26	1.97	0.59	1.88	1.35
South Africa	1.81	1.59	0.52	1.51	1.38
Europe	1.59	1.36	0.37	1.31	1.32
North America	1.59	1.35	0.42	1.28	1.37
South America	1.25	1.06	0.28	1.02	1.30
Oceania	0.09	0.06	0.02	0.07	1.36
Greenland	0.03	0.02	0.01	0.02	1.34
Antarctica	0.0021	0.0018	0.0005	0.0018	1.34

Not surprisingly, the atmospheric transport patterns of resuspended MP particles are relatively similar to those of mineral dust. For instance, a major export route of MPs from the Saharan desert into the Atlantic Ocean can be seen. The mean global lifetime of dust is about 4 days, based on Groot Zwaaftink et al.^29^ In our study, the mean global lifetime of MP spheres is 6 days, while for MP fibers, it is approximately 9 days. The longer lifetime of MPs is attributed to their lower density in comparison with mineral dust. Additionally, fibers have smaller settling velocities and thus reside longer in the atmosphere than spheres.^40^

The annual average resuspended MP near-surface concentrations in the atmosphere range up to 0.8 ng m^–3^ when assuming that the emitted MPs are fibers, with somewhat lower values for spheres reflecting their shorter residence time in the atmosphere (Figures 4a and S2a). The annual average fiber concentration is 9 (5–11) pg m^–3^ over North Africa and 20 (12–23) pg m^–3^ over West Asia (Table 2). Over the other continents, concentrations are much lower. Most of the resuspended MP mass is found in the lower troposphere, but transport to higher altitudes and even into the stratosphere occurs at low latitudes (Figures 4b and S2b). Fibers reach higher altitudes than spheres.

As shown in Figures 4c and S2c, the total deposition of MP fibers ranges up to 44,000 ng m^–2^ yr^–1^. The annual deposition is largest over West Asia with 30 (16–32) tonnes and over North Africa with 23 (13–26) tonnes (Table 3). With 75 (43–83) tonnes yr^–1^ deposited over land and 29 (18–33) tonnes yr^–1^ over oceans, we find that nearly 30% of the resuspended MP fibers reach the ocean. For spheres, relatively more deposition occurs over land [84 (49–93) tonnes yr^–1^] and relatively less over the oceans [20 (12–23) tonnes yr^–1^], again indicating their shorter atmospheric lifetime and reduced transport potential. However, both shapes are deposited globally, indicating that MPs can pose a threat even in remote regions. Small amounts are even deposited over Antarctica. Dry deposition is relatively more important for large particles in the source regions, while wet deposition is more significant for small particles transported over the oceans (Figure S3). Accumulation mode particles (100–1000 nm in diameter) are generally enriched in the deposition to the ocean relative to the deposition over land, with possible consequences for marine life that can easily ingest nanoplastic particles.^47^

Average MP near-surface concentrations in the layer 0–150 m (agl) are relatively variable throughout the year, with values (mean of spheres and fibers) of 0.60, 0.81, 1.02, and 0.57 pg m^–3^ in DJF, MAM, JJA, and SON, respectively (Figures S4 and S5). The vertical transport of MPs is strongest during JJA, whereas during DJF season, MPs are mostly found near the surface (Figures S4 and S5).

It is difficult to validate our model calculations because measurements of suspended and deposited MPs are almost completely missing in bare soil areas, and elsewhere, they are not specific to resuspended MPs. Furthermore, differences in the sampling, analysis techniques, and measurement periods render the comparison between these measurements and the model results difficult. Since the majority of the studies report atmospheric concentration and deposition in MP particle number counts, we convert the values to mass assuming a typical plastic density of 1050 kg m^–3^ and spherical particles with a diameter of 20 μm^48^ for concentration and 50 μm^49^ for deposition. For one measured MP particle per m^–3^ and per m^–2^, these values correspond to 4.4 ng m^–3^ and 0.07 μg m^–2^ for concentration and deposition, respectively.

Abbasi et al.^50^ measured the MP content in suspended dust in urban and industrial areas in southern Iran during August 2017. The majority of the sampled MPs were fibers of size from 2 to 100 μm and, with some particles larger than 100 μm. The average atmospheric concentration was about 0.7 MP m^–3^ or 3.1 ng m^–3^, which is higher than our simulated average concentration of 0.06 ng m^–3^ in this region, indicating plastic contamination from urban sources.

Abbasi and Turner^51^ found that total deposition is around 330 μg m^–2^ yr^–1^ (4750 MPs m^–2^ yr^–1^) in the remote Mount Derak region (Iran), quite higher than our flux of 4.5 μg m^–2^ yr^–1^, reflecting contributions from other plastic sources.

Tian et al.^27^ investigated the MPs in wind-blown dust from farmlands in northern China and found that, on average, 276 MPs m^–2^ day^–1^ or 6935 μg m^–2^ yr^–1^ were deposited in these regions, a value much higher than our estimation of 0.3 μg m^–2^ yr^–1^ in that region. This probably indicates the contribution of MPs from contaminated agricultural soils or other sources, possibly including primary MP sources.

When also considering measurements in regions where resuspended MPs are probably only a small fraction of total MP concentrations, Gaston et al.^52^ measured 24.6 ng m^–3^ in urban parts of California, while we estimate 0.0008 ng m^–3^ for the same region. Prata et al.^53^ reported an atmospheric concentration of 26 ng m^–3^ in the city of Aveiro (Portugal), a value remarkably higher than our 0.0002 ng m^–3^ estimate. Klein and Fischer^54^ found that the total deposition of MPs in the urban area of Hamburg was 19.3 μg m^–2^ day^–1^ or 7026 μg m^–2^ yr^–1^, compared to our value of 0.04 μg m^–2^ yr^–1^. Furthermore, Allen et al.^55^ reported that, on average, 25.6 μg m^–2^ day^–1^ or 9344 μg m^–2^ yr^–1^ are deposited in the remote area of the French Pyrenees, higher than our value of 0.2 μg m^–2^ yr^–1^.

Overall, it appears that our simulations show lower concentration and deposition values than the few measured ones published for bare soil, while we strongly underestimate measurements in urban areas, where it is expected that resuspended MP particles are only a minor fraction of the total MP abundance. However, it is also clear that the available measurements are too scarce to fully validate our model calculations, and the necessary conversion of number concentrations to mass concentrations adds uncertainty to the comparisons.

In this paper, we have presented a method to estimate the resuspension of MPs from bare soil regions and made a first estimate that should be revised when more measurements of the MP content in soils and their enrichment in resuspended dust become available. Resuspensions show a maximum during the northern hemisphere summer, which can favor MP vertical transport to higher altitudes in the troposphere and into the stratosphere. Simulations indicate that fibers can be dispersed more than equivalent volume spheres and reach remote regions. The comparison with atmospheric measurements suggests that resuspension from bare soils together with mineral dust is currently still a smaller source of atmospheric MPs than other MP sources. However, the broad distribution of this source also leads to global MP dispersion through the atmosphere, including remote environments. Since MPs accumulate in the environment, it is likely that the MP soil content and, thus, the resuspensions will increase in the future, increasing their importance compared to primary MP sources.

## Supporting Information Available

Figure for continental regions, figure for transport of spheres, figure for wet and dry deposition of fibers, figures for seasonal atmospheric concentration of spheres and fibers, table for MP soil mass fraction data, table for seasonal resuspensions, and tables for continental concentration and deposition of spheres (PDF)
