# Supplementary material for: Atmospheric Resuspension of Microplastics from Bare Soil Regions
Source: Environ Sci Technol. 2024 May 20;58(22):9741–9. doi: 10.1021/acs.est.4c01252 (PMC11155246; doi:10.1021/acs.est.4c01252)
Supplement: Supplementary file 1 — es4c01252_si_001.pdf [file es4c01252_si_001.pdf]

Supporting Information: "Atmospheric Resuspension of Microplastics from Bare Soil Regions."

Ioanna Evangelou,* Daria Tatsii, Silvia Bucci, and Andreas Stohl

Department of Meteorology and Geophysics, University of Vienna, Universitätsring 1, 1010, Vienna, Austria

E-mail: ioanna.evangelou@univie.ac.at

13 pages

Contents

Figure S1: Geographical regions used for continental calculations....S2

Figure S2: Transport of MP spheres....S3

Figure S3: Wet and dry deposition of fibers....S4

Figure S4: Seasonal atmospheric concentration and vertical profile for spheres....S5

Figure S5: Seasonal atmospheric concentration and vertical profile for fibers....S6

Table S1: Studies reporting the fraction of MP in bare soils....S7

Table S2: Total seasonal continental REC resuspensions....S8

Table S3: Annual mean MP concentrations for spheres over different continental regions....S9

Table S4: Annual total deposition for spheres over different continental regions....S10

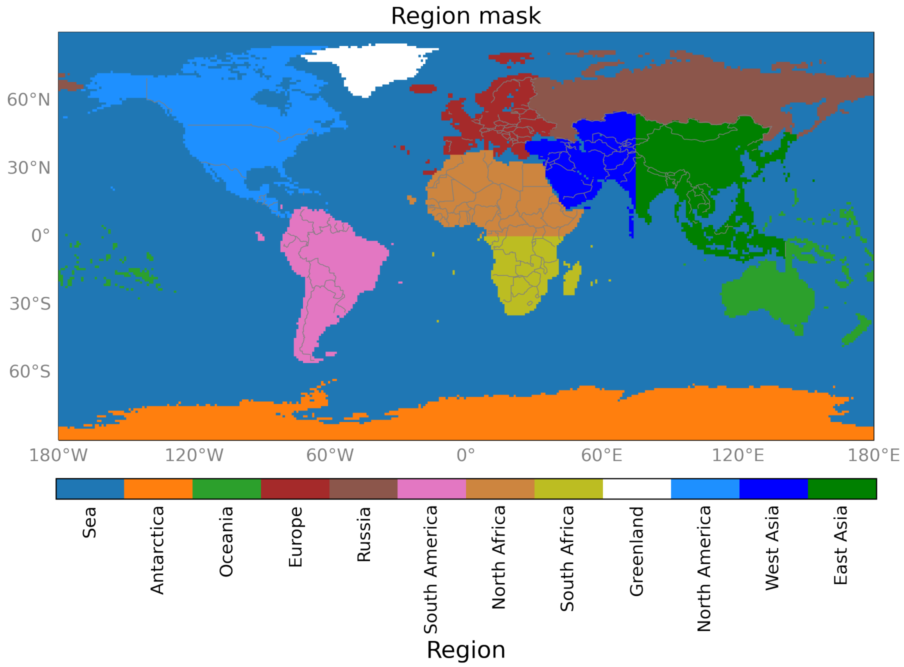

Figure S1: Geographical regions used for the continental resuspensions, atmospheric concentration and deposition.

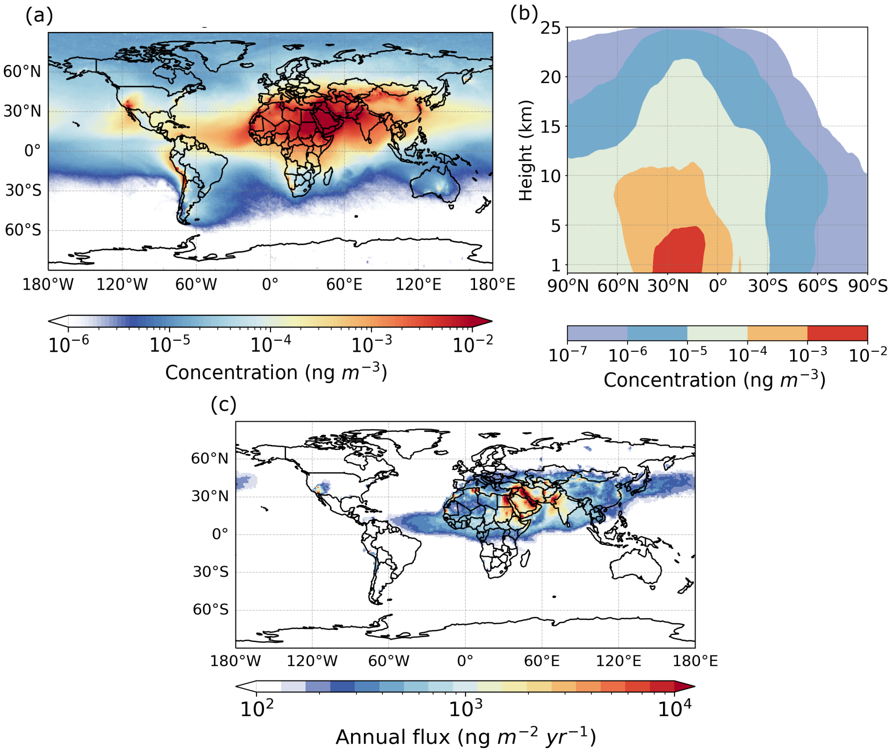

Figure S2: Transport of MPs. Atmospheric MP concentrations simulated by FLEXPART for the year 2018. (a) Near-surface concentrations (0-150 m agl) and (b) zonally averaged concentrations as a function of latitude and altitude when assuming that the emitted MP particles are spheres. (c) Sum of simulated wet and dry deposition fluxes of MPs for the year 2018 for spheres.

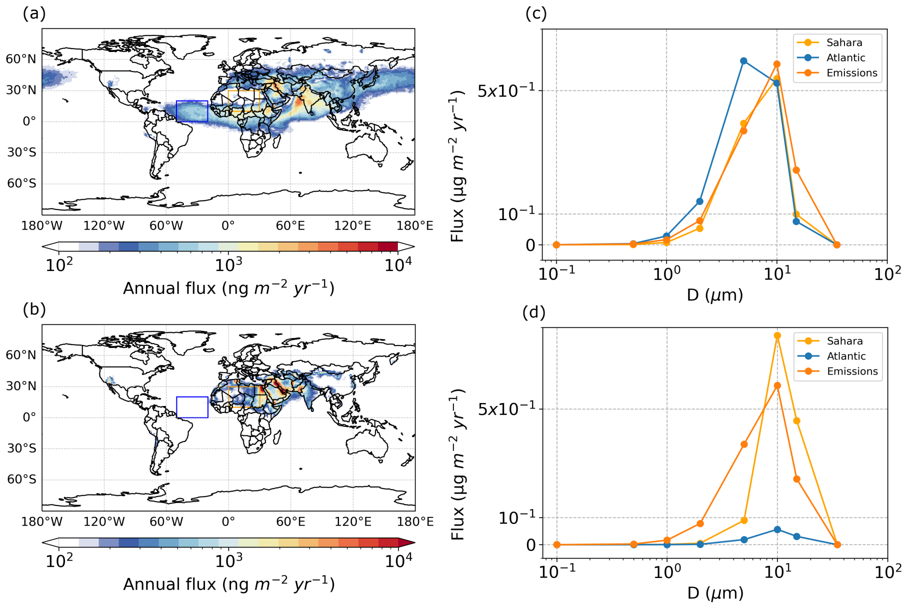

Figure S3: Wet (panel a) and dry deposition (panel b) for fibers. Size distribution of average wet deposition (panel c) and dry deposition (panel d) over part of Sahara and part of the Atlantic Ocean (orange and blue box respectively). The size distribution of the average resuspensions of the Sahara box is also depicted for comparison.

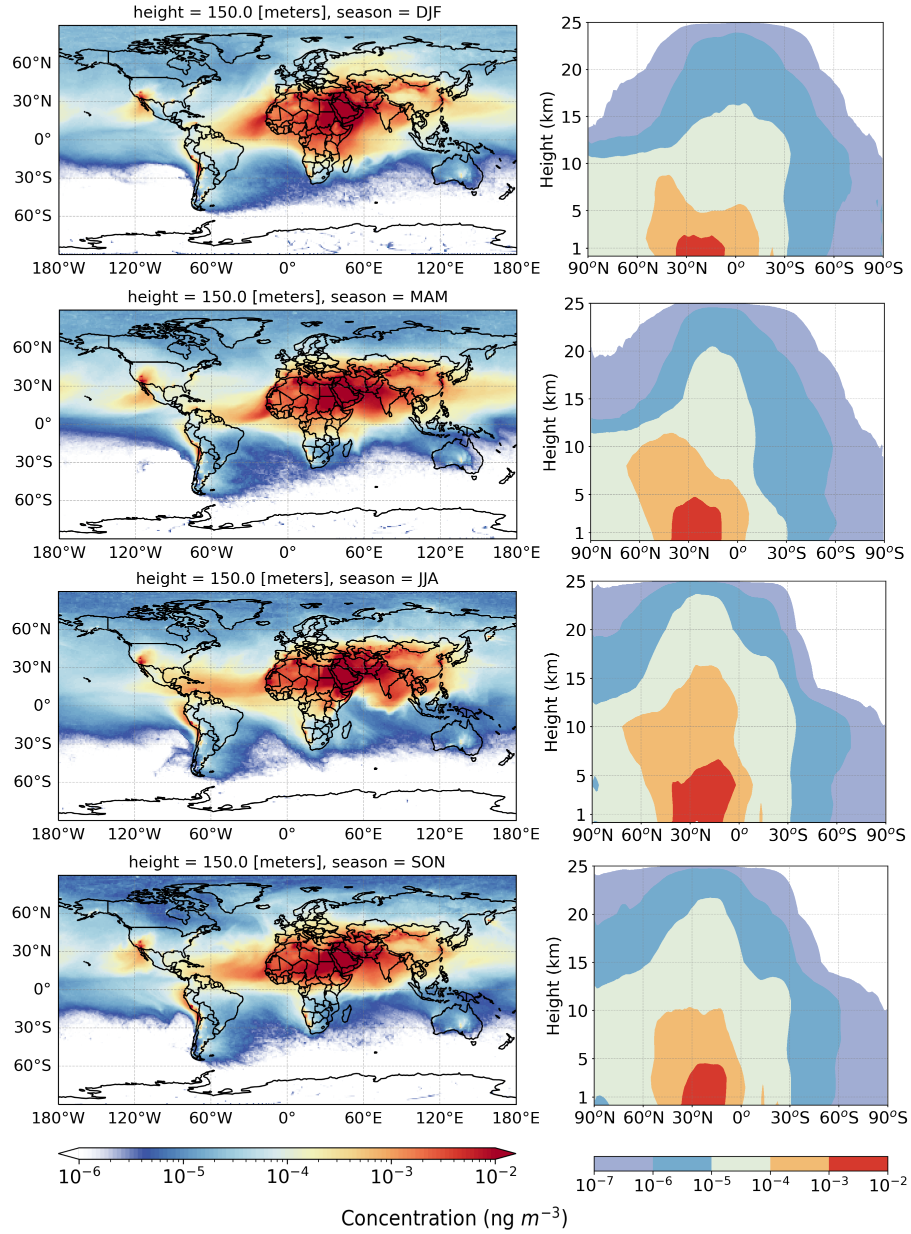

Figure S4: Seasonal average near-surface concentration (left column) and the corresponding latitudinal vertical profile (right column) for spheres.

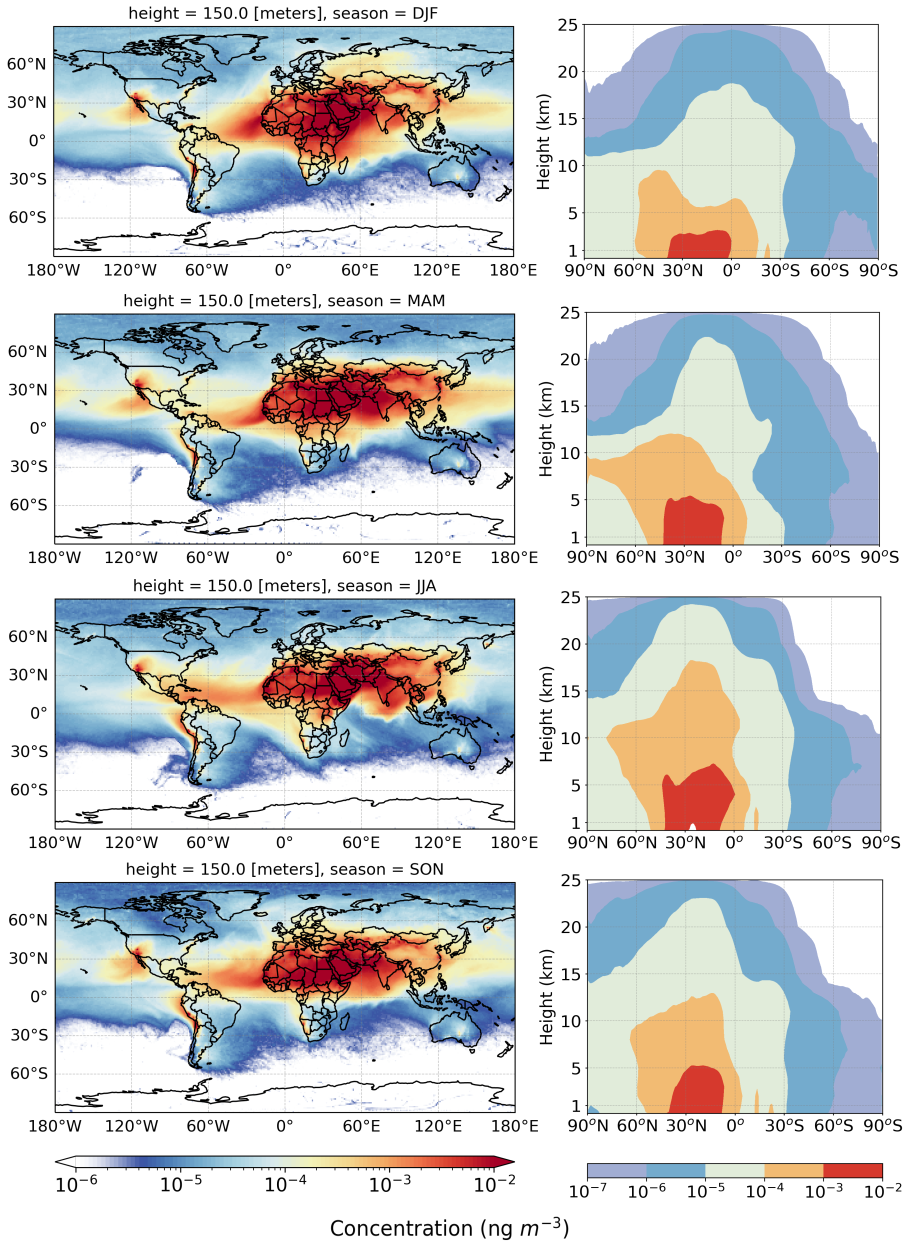

Figure S5: Same as Figure 4 but when assuming that the emitted particles are fibers.

Table S1: Studies reporting the fraction of MP in bare soils across the world, the shape and size used for MP items to MP mass conversion and the corresponding land use. If no conversion was needed, size and shape are not reported.

Study	Region	Mass fraction ($\mathrm{mg\,kg}^{-1}_{dry\,soil}$)	Shape and Size	Land use
Abbasi et al., 2021[1]	Iran	0.001	fiber, 100 μm	Desert
Álvarez-Lopeztello et al., 2021[2]	Mexico	0.43	fiber, 325 μm	Savanna
Ding et al., 2021[3]	China	35.0	sphere, 250 μm	Sand
Feng et al., 2023[4]	China	0.06	sphere, 50 and 100 μm	Vacant land
Feng et al., 2023[5]	China	0.003	sphere, 60 μm	Slope grassland
Liu et al., 2023[6]	China	2.4	fiber, 150 μm	Undisturbed soil
Rezaei et al., 2019[7]	Iran	0.46		Rangeland
Rolf et al., 2022[8]	Germany	5.9	sphere, 70 μm	Floodplain
Scheurer and Bigalke, 2018[9]	Switzerland	6.2		Floodplain
Wang et al., 2021[10]	China	0.03	sphere, 232 μm	Desert
Weber and Opp, 2020[11]	Germany	12.8	sphere, 2000 μm	Floodplain
Yang et al., 2022[12]	China	0.003	fiber, 200 μm	Non-exploited soil
Zhang and Liu, 2018[13]	China	0.7	fiber, 150 μm	Riparian soil
Zhang et al., 2022[14]	China	29.15		Desert
Zhang et al., 2023[15]	Uzbekistan	0.07	sphere, 40 μm	Dry land
Zhou et al., 2019[16]	China	13.7	sphere, 50 μm	Vacant land
Zhou et al., 2021[17]	China	0.26	sphere, 50 μm	Riparian soil

Table S2: Total continental REC resuspensions for December, January and February (DJF), for March, April, May (MAM), for June, July and August (JJA),and for September, October, November (SON).

Region	DJF		MAM		JJA		SON	
				tonnes				
total global land surface	23.85	(12.39,1.52)[a]	27.81	(19.59,1.52)	43.60	(29.29,1.50)	19.92	(11.10,1.52)
West Asia	10.91	(5.03,1.51)	13.28	(9.06,1.54)	29.29	(18.88,1.52)	8.91	(4.91,1.56)
North Africa	11.10	(6.14,1.54)	11.98	(8.24,1.51)	12.64	(8.57,1.51)	9.13	(4.85,1.51)
East Asia	0.76	(0.44,1.59)	1.559	(1.25,1.61)	0.87	(0.65,1.57)	0.78	(0.51,1.62)
North America	0.50	(0.35,1.57)	0.65	(0.66,1.53)	0.34	(0.67,1.74)	0.38	(0.31,1.56)
South America	0.39	(0.23,2.25)	0.16	(0.14,2.26)	0.32	(0.24,1.85)	0.50	(0.31,1.93)
Europe	0.11	(0.06,1.52)	0.16	(0.10,1.51)	0.08	(0.07,1.56)	0.07	(0.041.53)
South Africa	0.05	(0.03,2.21)	0.02	(0.02,2.16)	0.04	(0.04,2.15)	0.07	(0.04,2.15)
Russia	0.011	(0.004,2.26)	0.006	(0.006,2.28)	0.020	(0.016,1.97)	0.058	(0.031,1.86)
Oceania	0.019	(0.006,1.76)	0.007	(0.003,1.76)	0.008	(0.004,1.78)	0.016	(0.006,1.77)
Greenland	0.000 09	(0.00004,1.43)	0.000 01	(0.00001,1.48)	0.0003	(0.0002,1.65)	0.0004	(0.0003,1.61)

[a] (geometric mean, geometric standard deviation)

Table S3: Annual mean MP near-surface (0-150 m agl) concentrations averaged over different continental regions when assuming that the emitted particles are spheres. REC concentrations as well as the MC mean and the standard deviation, the geometric mean and geometric standard deviation are presented.

Region	REC[a]	$\overline{x}$	σ	μ_g	σ_g
	pg m^{-3}				
West Asia	13.9	12.1	4.2	11.4	1.41
North Africa	6.3	5.7	2.2	5.4	1.45
East Asia	1.0	0.9	0.3	0.9	1.39
North America	0.10	0.09	0.03	0.09	1.38
South America	0.22	0.19	0.05	0.18	1.31
Europe	0.27	0.24	0.07	0.23	1.35
South Africa	0.28	0.26	0.09	0.24	1.40
Russia	0.07	0.07	0.02	0.06	1.37
Oceania	0.013	0.012	0.004	0.011	1.38
Greenland	0.018	0.017	0.007	0.015	1.54
Antarctica	0.0004	0.0004	0.0002	0.0003	1.51

[a] Reference Emission Case

Table S4: Same as Table S3 but for annual total (wet and dry) deposition.

Region	REC	$\overline{x}$	σ	μ_g	σ_g
	tonnes				
total global land surface	83.9	70.9	23.2	67.3	1.38
total global ocean surface	20.1	17.7	5.8	16.8	1.39
West Asia	39.1	32.3	10.6	30.6	1.39
North Africa	27.3	23.4	8.3	22.0	1.42
East Asia	10.7	9.4	3.0	8.9	1.37
Russia	1.63	1.43	0.43	1.37	1.35
North America	1.57	1.33	0.41	1.27	1.36
Europe	1.29	1.10	0.30	1.06	1.32
South America	1.17	0.99	0.25	0.96	1.29
South Africa	1.05	0.93	0.30	0.88	1.37
Oceania	0.07	0.06	0.02	0.06	1.35
Greenland	0.02	0.03	0.01	0.02	1.35
Antarctica	0.0017	0.0015	0.0004	0.0014	1.36

References

(1) Abbasi, S.; Turner, A.; Hoseini, M.; Amiri, H. Microplastics in the Lut and Kavir Deserts, Iran. *Environmental Science and Technology* **2021**, *55*, 5993–6000.

(2) Álvarez-Lopeztello, J.; Robles, C.; del Castillo, R. F. Microplastic pollution in neotropical rainforest, savanna, pine plantations, and pasture soils in lowland areas of Oaxaca, Mexico: Preliminary results. *Ecological Indicators* **2021**, *121*.

(3) Ding, L.; Wang, X.; Ouyang, Z.; Chen, Y.; Wang, X.; Liu, D.; Liu, S.; Yang, X.; Jia, H.; Guo, X. The occurrence of microplastic in Mu Us Sand Land soils in northwest China: Different soil types, vegetation cover and restoration years. *Journal of Hazardous Materials* **2021**, *403*, 123982.

(4) Feng, S.; Lu, H.; Yao, T.; Tang, M.; Yin, C. Analysis of microplastics in soils on the high-altitude area of the Tibetan Plateau: Multiple environmental factors. *Science of the Total Environment* **2023**, *857*, 159399.

(5) Feng, S.; Lu, H.; Yao, T. Effects of multiple environmental factors and land use patterns on microplastic distribution in the topsoil of the Qinghai and Sichuan provinces of China. *Journal of Environmental Chemical Engineering* **2023**, *11*, 109657.

(6) Liu, X.; Cheng, P.; Zhou, J.; Fan, Y.; Fu, Y.; Tan, L.; Lan, J.; Zhang, L.; Gu, H.; Bi, Y. Microplastic Characteristics in Equus kiang (Tibetan Wild Ass) Feces and Soil on the Southern Tibetan Plateau, China. *Environmental Science and Technology* **2023**, *57*, 9732–9743.

(7) Rezaei, M.; Riksen, M. J.; Sirjani, E.; Sameni, A.; Geissen, V. Wind erosion as a driver for transport of light density microplastics. *Science of the Total Environment* **2019**, *669*, 273–281.

(8) Rolf, M.; Laermanns, H.; Kienzler, L.; Pohl, C.; Möller, J. N.; Laforsch, C.; Löder, M. G.; Bogner, C. Flooding frequency and floodplain topography determine abundance of microplastics in an alluvial Rhine soil. *Science of the Total Environment* **2022**, *836*.

(9) Scheurer, M.; Bigalke, M. Microplastics in Swiss Floodplain Soils. *Environmental Science and Technology* **2018**, *52*, 3591–3598.

(10) Wang, F.; Lai, Z.; Peng, G.; Luo, L.; Liu, K.; Huang, X.; Xu, Y.; Shen, Q.; Li, D. Microplastic abundance and distribution in a Central Asian desert. *Science of the Total Environment* **2021**, *800*, 149529.

(11) Weber, C. J.; Opp, C. Spatial patterns of mesoplastics and coarse microplastics in floodplain soils as resulting from land use and fluvial processes. *Environmental Pollution* **2020**, *267*, 115390.

(12) Yang, L.; Kang, S.; Wang, Z.; Luo, X.; Guo, J.; Gao, T.; Chen, P.; Yang, C.; Zhang, Y. Microplastic characteristic in the soil across the Tibetan Plateau. *Science of the Total Environment* **2022**, *828*, 154518.

(13) Zhang, G. S.; Liu, Y. F. The distribution of microplastics in soil aggregate fractions in southwestern China. *Science of the Total Environment* **2018**, *642*, 12–20.

(14) Zhang, Z.; Guo, J.; Wang, P. Occurrence, sources, and relationships of soil microplastics with adsorbed heavy metals in the Ebinur Lake Basin, Northwest China. *Journal of Arid Land* **2022**, *14*, 910–924.

(15) Zhang, P.; Wang, J.; Huang, L.; He, M.; Yang, H.; Song, G.; Zhao, J.; Li, X. Microplastic transport during desertification in drylands: Abundance and characterization of soil microplastics in the Amu Darya-Aral Sea basin, Central Asia. *Journal of Environmental Management* **2023**, *348*, 119353.

(16) Zhou, Y.; Liu, X.; Wang, J. Characterization of microplastics and the association of heavy metals with microplastics in suburban soil of central China. *Science of the Total Environment* **2019**, *694*, 133798.

(17) Zhou, Y.; He, G.; Jiang, X.; Yao, L.; Ouyang, L.; Liu, X.; Liu, W.; Liu, Y. Microplastic contamination is ubiquitous in riparian soils and strongly related to elevation, precipitation and population density. *Journal of Hazardous Materials* **2021**, *411*, 125178.
